# Emerging Protein Sources for Food Production and Human Nutrition

**DOI:** 10.3390/molecules28062676

**Published:** 2023-03-16

**Authors:** Przemysław Łukasz Kowalczewski, Anubhav Pratap-Singh, David D. Kitts

**Affiliations:** 1 Department of Food Technology of Plant Origin, Faculty of Food Science and Nutrition, Poznań University of Life Sciences, 60-637 Poznań, Poland; 2 Food, Nutrition, and Health, Faculty of Land & Food Systems, The University of British Columbia, 2205 East Mall, Vancouver, BC V6T 1Z4, Canada; david.kitts@ubc.ca

## 1. Introduction

It is estimated that by 2050, the world’s population will be up to 9 billion. Feeding the rising population with the appropriate amounts of food, and in particular, with adequate protein supply is an emergent focus of current research activities in the field of food science. Global environmental changes, rapidly changing socio-economic conditions, and geopolitical issues endanger food sustainability, and generate the need to search for new, unconventional sources of protein for human nutrition. Considering the above-mentioned worldwide circumstances, many scientists around the world are dealing with the subject of alternative protein sources, as well as examining their properties and safety. Ensuring an adequate supply of protein, but also its appropriate quality and nutritional value, is one of the main challenges facing the world of science.

With this mission in mind, the scope of this Special Issue of Molecules, entitled “Emerging Protein Sources for Food Production and Human Nutrition”, is devoted to the latest advances in analytics and the application of new methods in the processing of proteins, both plant and animal, as well as methods of production and testing foods enriched with these proteins. In this SI, 10 original research manuscripts were collected and published. The articles included in this collection are briefly described below.

## 2. Plant-Based Protein Sources

Miedzianka et al. analyzed the effect of the acetylation of plant proteins on their functional and nutritional properties. In their first paper, an attempt was made to increase the value of rice protein concentrate [1] by improving the properties of a commercial protein preparation. After the acetylation process, the influence of this chemical modification on the chemical composition, digestibility and protein modeling was analyzed using SDS-PAGE, electrophoresis and FT-IR spectroscopy. Electrophoresis showed that the content of the main fractions of rice proteins (prolamine and glutelin) decreased with the increase in the concentration of the modifying reagent. Using spectroscopic analysis, wavenumbers corresponding to the presence of proteins or lipids, aromatic systems and carbohydrates were observed. However, the use of acetic anhydride did not significantly affect the digestibility of the modified rice protein concentrate, while acetylation resulted in a significant increase in its emulsifying properties and water-binding capacity. A slight increase in protein solubility and a decrease in foaming capacity were also observed in the modified rice protein concentrate.

In the second publication, the effect of acetylation with different doses of acetic anhydride on the chemical composition and selected functional properties of a commercial pumpkin protein concentrate was determined [2]. The electrophoretic analysis showed that in the acetylated pumpkin protein, the content of the heaviest protein (with a molecular weight of 35 kDa) decreased with the increase in concentration of the modifying reagent. The acetylation of pumpkin protein caused a significant increase in the water-binding capacity, oil absorption and emulsifying properties that were already at a dose of 0.4 mL/g. In addition, an increase in the foaming capacity of preparations obtained with 2.0 mL/g of acetic anhydride was demonstrated, while the acetylation of 0.4 and 1.0 mL/g caused a decrease in protein solubility compared to native pumpkin protein. It can, therefore, be concluded that acetylation improves the functional properties of commercial protein preparations, which broadens the possibilities of their use in the food industry.

Potato protein is considered to be one of the most valuable plant-based proteins due to the high content of essential amino acids and biological activity [3]. Numerous literature data indicate, however, that the enzymatic hydrolysis of proteins that has been properly carried out can significantly improve both the nutritional value and biological activity. The study of Kowalczewski et al. [4] is a report on the effect of the enzymatic hydrolysis of potato juice proteins, combined with membrane filtration. The obtained hydrolysate was characterized in terms of nutritional value and biological activity, including the evaluation of the amino acid profile and score, the content of mineral compounds, as well as antioxidant and cytotoxic activity in vitro. It was also found that the enzymatic hydrolysis of potato juice in the reactor with the ultrafiltration membrane separation system increased the cytotoxic activity of the processed material. IC_50_ toxic doses of the hydrolysate for cancer cells were significantly lower than those of fresh potato juice. Moreover, IC_50_ toxic doses of the concentrate were lower for cancer cells than for normal cells. Cytotoxicity against the human gastric cancer cell line (Hs 746T), the human colon cancer cell line (Caco-2), the human colorectal adenocarcinoma cell line (HT-29) and the normal human colon mucosa cell line (CCD 841 CoN) showed cytotoxic activity specifically directed against cancer cells. Therefore, it can be concluded that the membrane filtration-assisted enzymatic hydrolysis of potato juice proteins may increase their biological activity and allow for potato juice to be used in the production of medicinal preparations.

## 3. Whey and Insects as a Protein Source

Another important article in the Special Issue is that of Smułek et al. [5] on hemp-seed-oil-based emulsions. The protein used to stabilize the emulsions was whey protein, which is an important raw material in the food industry. A novel solution proposed by the authors of the paper was the addition of an extract from *Aesculus hippocastanom*, which contains natural compounds—for example, saponins, as a co-surfactant. The coexistence of these two emulsifiers made it possible to obtain stable emulsions with an average droplet size of 200–300 nm. Moreover, the obtained emulsions were characterized by good rheological properties. In addition, microbiological studies showed that the tested emulsion systems positively influenced the activity of a probiotic strain of the genus *Lactobacillus*. Thus, the described emulsions represent a good solution with high application values.

Edible insects are commonly used as food in many parts of the world, mainly in Africa, Latin America and Asia [6]. According to the FAO, more than 1900 different species of insects are eaten worldwide. These include mealworms and crickets [7].

Dion-Poulin et al. [8], in their paper, described studies on the evaluation of the functional properties of two commercial insect meals (obtained from *Gryllodes sigillatus* and *Tenebrio molitor*) and their respective hydrolysates obtained using Alcalase^®^, conventionally and after the pressurization pretreatment of insect powders. It was observed that water-binding capacity, foaming and gelling properties were not improved after enzymatic hydrolysis. The pre-pressure treatment of mealworm meals probably caused protein denaturation and aggregation, which reduced the degree of hydrolysis. As expected, enzymatic digestion (with and without pressure) increased the solubility, reaching values close to 100%. The pre-pressure treatment of mealworm flour further improved its solubility compared to the control hydrolysate, while pressure treatment reduced the solubility of cricket meals. The oil-binding capacity was also improved after enzymatic hydrolysis.

In the research of Boukil et al. [9], pressure treatment was also used. The ability of high hydrostatic pressure (HHP) in combination with enzymatic hydrolysis by Alcalase^®^ or pepsin has been studied to improve the in vitro digestion of mealworm proteins, particularly allergy-causing proteins. The effect of the in vitro digestion of the main allergenic proteins of mealybug was enhanced by the use of HPP; therefore, HHP-assisted enzymatic hydrolysis is an alternative strategy to conventional hydrolysis to produce a large amount of peptide derived from allergenic insect proteins and reduce their immunoreactivity in food, nutraceuticals and pharmaceuticals.

The analysis of the properties of mealworm proteins was also undertaken by a group of scientists led by Gravel [10]. The effect of defatting powder from mealworm (*Tenebrio molitor*) with hexane on protein profiles and the techno-functionality of the obtained preparations was analyzed. Major protein profiles were shown to be similar between hexane defatted and non-defatted samples; however, some specific differences in content (e.g., hexamerin 2) were observed and characterized using proteomic tools. Protein solubility was significantly lower in the case of *Tenebrio molitor* meals in comparison to protein extracts defatted with hexane. A significant increase in the foaming capacity of the defatted fractions was also observed.

Consumer acceptance of insects as food is a necessary step to expand their presence in the market. The most popular way to eat insects in Europe is to use them to enrich traditional products. Zielińska and Pankiewicz [11] studied the characteristics of shortcake biscuits enriched with *Tenebrio molitor* flour and they examined properties such as nutrient composition, color, physical and antioxidant properties, starch digestibility and in vitro glycemic index. They showed that the substitution of wheat flour with mealworm flour changed the nutritional value of the products—a progressive increase in the protein and ash contents of biscuits as the concentration of mealworm flour increased. Moreover, mealworms were found to have high antioxidant potential, as evident from the higher free-radical scavenging activity of biscuits enriched in mealworm flour compared to the control. Additionally, the supplementation of mealworm flour to biscuits caused an increase in slowly digested starch, with a decrease in rapidly digested starch. This is important information for consumers because the dietary benefits attributed to SDS are associated with a slower postprandial rise in blood glucose and glycemia maintenance for longer periods compared to RDS, which results in a rapid rise and then a rapid fall in blood glucose. Thus, the authors emphasized that edible insects are a source of valuable nutrients and manifest health-promoting properties; thus, using them in designing health-promoting foods seems to be justified.

A group of researchers led by Smarzyński [12] analyzed the impact of using cricket powder (CP) on the molecular properties of water in model shortcake biscuits, and characterized their nutritional properties. The partial replacement of wheat flour with CP in biscuits increased their nutritional value, but also affected the analyzed physical properties. In addition, a small addition of CP improved the taste, texture, appearance and overall attractiveness ratings of the biscuits. Changes in the physical properties of the biscuits were also observed. The more wheat flour was replaced by CP, the lower the hardness of the biscuits. Analysis of the molecular dynamics of water, measured by LF NMR, indicated a decrease in the value of the short components of the spin–spin (T_21_) relaxation times, which indicates a decrease in the dynamics of water molecules bound to the polymer matrix.

The analysis of the impact of the use of CP in another product—gluten-free bread—was also dealt with by a group of scientists led by Kowalczewski [13]. The nutritional value as well as antioxidant and β-glucuronidase activities were assessed after the simulated in vitro digestion of gluten-free breads enriched with 2%, 6% and 10% of house cricket (*Acheta domesticus*) powder. The addition of CP significantly increased the nutritional value, both in terms of protein and, above all, minerals. A significant increase in the content of polyphenolic compounds and antioxidant activity in enriched bread was also demonstrated. The use of CP also reduced the undesirable activity of β-glucuronidase by 65.9% (compared to the control bread) in the small intestine, and by as much as 78.9% in the large intestine. The effect of bread on the intestinal microflora was also assessed and no inhibitory effect on the growth of the intestinal microflora (Bifidobacterium and Lactobacillus) was found. The presented results indicate the benefits of using CP to increase the nutritional value and biological activity of gluten-free food products.

## Data Availability

Not applicable.
